# Association of mitochondrial oxidative capacity with physical fitness in ageing: the Baltimore longitudinal study of ageing

**DOI:** 10.1093/ageing/afag022

**Published:** 2026-02-06

**Authors:** Caterina Trevisan, Qu Tian, Kenneth W Fishbein, Sarah Church, Eleanor M Simonsick, Josephine M Egan, Stefano Volpato, Luigi Ferrucci

**Affiliations:** Department of Medical Sciences, University of Ferrara, Ferrara, Emilia-Romagna, Italy; Aging Research Center, Department of Neurobiology, Care Sciences and Society, Karolinska Institutet and Stockholm University, Stockholm, Sweden; Longitudinal Studies Section (LSS), Translational Gerontology Branch, Intramural Research Program, National Institute on Aging, National Institutes of Health, Baltimore, Maryland, USA; Laboratory of Clinical Investigation, Intramural Research Program, National Institute on Aging, National Institutes of Health, Baltimore, Maryland, USA; Clinical Research Core (CRC), National Institute on Aging, National Institutes of Health, Baltimore, Maryland, USA; Clinical Research Core (CRC), National Institute on Aging, National Institutes of Health, Baltimore, Maryland, USA; Longitudinal Studies Section (LSS), Translational Gerontology Branch, Intramural Research Program, National Institute on Aging, National Institutes of Health, Baltimore, Maryland, USA; Laboratory of Clinical Investigation, Intramural Research Program, National Institute on Aging, National Institutes of Health, Baltimore, Maryland, USA; Clinical Research Core (CRC), National Institute on Aging, National Institutes of Health, Baltimore, Maryland, USA; Department of Medical Sciences, University of Ferrara, Ferrara, Emilia-Romagna, Italy; Longitudinal Studies Section (LSS), Translational Gerontology Branch, Intramural Research Program, National Institute on Aging, National Institutes of Health, Baltimore, Maryland, USA

**Keywords:** ageing, mitochondria, energetic cost of walking, physical fitness, cohort study, older people

## Abstract

**Background:**

In younger individuals, fitness is mostly influenced by muscle mitochondrial oxidative phosphorylation (OxPhos) and cardiac output. However, compared with younger individuals, various impairments may also negatively affect fitness in older adults.

**Objective:**

To investigate the relationship of OxPhos with cardiorespiratory fitness, the energetic cost of walking and aerobic resilience with respect to age.

**Design:**

Cross-sectional.

**Setting:**

Population.

**Subjects:**

Six hundred and forty-nine Baltimore longitudinal study of ageing participants (mean age 64.5 years, 56.9% females).

**Methods:**

Muscle mitochondrial OxPhos was measured as phosphocreatine recovery rate (k_PCr_) through ^31^P magnetic resonance spectroscopy. Based on age- and sex-specific k_PCr_ z-scores, we classified individuals with low (≤ −0.5 standard deviations [SD]), average (−0.5 to 0.5SD) and high (>0.5SD) OxPhos. Cardiorespiratory fitness was measured as peak oxygen consumption (MVO_2_ peak) during a treadmill testing. The energetic cost of usual pace walking was expressed as the average oxygen consumption per 100 metres. Aerobic resilience was the ratio between MVO_2_ peak and average VO_2_ during usual pace walking.

**Results:**

Participants with higher k_PCr_ had 4.07 (95%CI: 2.88, 5.26) ml/kg/min higher MVO_2_ peak and 0.19 (95%CI: 0.06, 0.32) higher aerobic resilience than those with lower k_PCr_. The energetic cost of walking was greater by 0.84 (95% CI: 0.21, 1.47) ml/kg/100 m in those with high than low k_PCr_. A multiplicative interaction between age and k_PCr_ was identified in the regressions predicting MVO_2_ peak and aerobic resilience (*p_interaction_* = 0.01), with differences between OxPhos groups attenuating after age 70.

**Conclusion:**

Muscle mitochondrial OxPhos contributes to interindividual variability in cardiorespiratory fitness, especially in young and middle adulthood.

## Key Points

- Higher muscle mitochondrial oxidative phosphorylation (OxPhos) is linked to greater cardiorespiratory fitness.
- Age modifies the association of OxPhos with cardiorespiratory fitness.
- OxPhos affects cardiorespiratory fitness more in younger than older adults.
- Adults with 1-SD lower OxPhos have cardiorespiratory fitness comparable to that of individuals 20 years older.

## Introduction

The ageing process is widely heterogeneous, and older adults experience a vast range of changes in health and functional characteristics [1, 2]. Interestingly, respiratory and muscle function, two fundamental contributors to physical performance, have the broadest heterogeneity range in older individuals [1]. Detecting the factors that influence the heterogeneity of physical performance in old age is crucial for identifying new targets of intervention to maintain functional ability and achieve healthy ageing [3]. However, evidence on this topic has not yet been fully clarified.

Among other roles, mitochondria provide most of the energy used by cells to perform various biological processes, and their function is highly correlated with physical and cognitive performance [4, 5]. Mitochondrial volume and oxidative phosphorylation (OxPhos) gradually decline with ageing [6, 7]. However, changes in mitochondrial OxPhos with ageing depend on several factors, including the level of physical activity [8], acute and chronic diseases and even subtle systemic dysfunctions that disrupt the homeostatic balance of the organism [9, 10]. Ultimately, these changes may alter cardiorespiratory fitness and energy metabolism [11, 12], resulting in impaired physical performance, including reduced muscle strength [11, 13], slower walking speed [4, 13, 14] and greater fatigability [12, 15].

Cardiorespiratory fitness is typically assessed through a graded exercise test to exhaustion, representing the actual aerobic capacity of fit, healthy individuals. In the general population, cardiorespiratory fitness is often approximated by peak oxygen consumption (MVO_2_ peak), defined as the highest rate of oxygen uptake achieved during a stress test when individuals may stop due to fatigue or other factors before reaching their maximal capacity [9]. Cardiorespiratory fitness depends on cardiovascular function and peripheral oxygen utilization, which are influenced mainly by parenchymal volume, perfusion and mitochondrial OxPhos. However, particularly in older persons, other factors, including musculoskeletal problems, pain, respiratory function and neurologic impairments [16], may also influence the level of effort an individual can afford and, consequently, MVO_2_ peak.

In this analysis, we examined whether the relationship of muscle mitochondrial OxPhos with fitness measures, including MVO_2_ peak, the energetic cost of walking, and aerobic resilience (i.e. the ratio between MVO_2_ peak and the average VO_2_ during walking at usual pace), would change with ageing.

## Methods

### Study population

This study uses data from participants of the Baltimore longitudinal study of ageing (BLSA), a prospective study started in 1958 and currently conducted by the National Institute on Aging (NIA) Intramural Research Program [17, 18]. The BLSA study protocol was approved by the Institutional Review Board of the Intramural Research Program of the National Institutes of Health (03AG0325: IRP/NIA/NIH); written informed consent was obtained from all participants at each visit. In brief, community-dwelling individuals aged 20 years and older and free from major chronic diseases and functional or cognitive deficits are enrolled on a voluntary basis. After the baseline evaluation, participants are reassessed every 4, 2, or 1 year if aged <60, 60–79, and 80+ years, respectively.

In the study, we examined a cross-sectional sample at the time of the first muscle mitochondrial OxPhos assessment (since April 2013). From the initial sample of 795 participants who underwent ^31^P magnetic resonance spectroscopy (MRS), we excluded 80 individuals with no available data on cardiorespiratory fitness and 66 who did not have a valid ^31^P MRS test for OxPhos measurement (details can be found below), resulting in a final analytical sample of 649.

### Data collection

Participant assessments were conducted by trained personnel using standardized protocols, which included administration of validated scales and questionnaires, personal interviews, physical examinations and laboratory and imaging tests. For the present study, we collected *sociodemographic, lifestyle* and *medical information* (detailed in **Appendix S1**  **in Supplementary Data**) along with the data described below.

#### Mitochondrial oxidative capacity

Mitochondrial OxPhos was assessed by the phosphocreatine (PCr) recovery rate. Briefly, participants underwent ^31^P MRS scanning of the quadriceps muscles of the left leg using a 3 T Achieva MRI scanner (Philips, Best, The Netherlands) to quantify phosphorous-containing metabolites [19]. For details, please see **Appendix S2**  **in Supplementary Data**. In this study, participants were categorised according to mitochondrial OxPhos. Since the current literature shows that mitochondrial OxPhos varies by age and sex, we computed z-scores of k_PCr_ by sex and age (considering the following categories: ≤55, 56–60, 61–65, 66–70, 71–75, 76–80, 81–85, 86+). Consequently, as shown in **Appendix S3**, participants were classified into three groups: lower mitochondrial OxPhos (k_PCr_ z-scores ≤ −0.5 standard deviation [SD]), average OxPhos (k_PCr_ z-scores between −0.5 and + 0.5 SD) and higher OxPhos (k_PCr_ z-scores >0.5 SD). The value of 0.5 SD is generally used as a cut-off to define a medium effect size of standardized mean differences [20].

#### Energetic cost of walking

Energy expenditure during walking was measured during a 2.5 minute walking test at the usual pace over a 20-metre course in an uncarpeted corridor. Participants were instructed to walk the course continuously for 2.5 minutes at their preferred pace. During the test, participants wore a portable indirect calorimeter (Cosmed K5, Cosmed, Rome, Italy) that monitored and recorded their average oxygen consumption and carbon dioxide production for each 30-s interval through breath-by-breath measures [21]. The total distance walked during the test was also recorded. In line with previous studies [21, 22], the average VO_2_ per kilogramme of body weight per minute (ml/kg/min), expressing the mean oxygen consumption during walking, was derived after excluding the first 1.5 minutes of the test in order to estimate the VO_2_ once the participant had reached a metabolic steady state. To obtain the energetic cost of walking at the usual speed, we computed the average oxygen consumption per metre walked (ml/kg/m) using the following formula: VO_2_ (ml/kg/min)^*^2.5 min/total distance (m). From this value, in order to facilitate data interpretation, we derived the average oxygen consumption per 100 metres walked (ml/kg/100 m).

#### Cardiorespiratory fitness

Treadmill testing for measurements of MVO_2_ peak was performed on all participants without high-risk cardiovascular diseases (e.g. moderate to severe aortic stenosis, ischemic heart diseases, or high-grade AV block). Participants underwent a modified Balke protocol in a motor-driven treadmill with constant speed (3.5 mph for men and 3.0 mph for women) and a slope that, starting from 0%, increased by 3% every 2 min. The test continued until participant exhaustion or symptom onset. During treadmill testing, VO_2_ (expressed in ml/kg/min) was measured by indirect calorimetry in participants wearing two-way non-rebreathing masks through the Vyntus CPX system (Jaeger Medical America, Inc., Moreno Valley, California, USA). VO_2_ assessment was performed every 30 seconds, and the highest value recorded during the test (MVO_2_ peak) was used as an indicator of individual aerobic capacity and cardiorespiratory fitness [23].

#### Aerobic resilience

The ratio between the MVO_2_ peak and the average VO_2_ during walking at usual pace (both measures expressed as ml/kg/min) was computed as an indicator of aerobic resilience. This parameter assesses how much the individual can increase their aerobic metabolism from usual activities to maximal effort. Higher values indicate that the individuals can perform a level of effort that requires an energy cost substantially higher than that of standard walking, while a lower value means that the energy cost of maximal effort is close or equal to the effort required during standard walking.

### Statistical analysis

Comparison of participants’ characteristics by mitochondrial OxPhos was performed using ANOVA or Chi-squared tests, as appropriate.

Relationships between mitochondrial OxPhos and fitness-related parameters were tested using simple correlation and expressed as Pearson r coefficients. Associations of energetic parameters with age were tested by linear regression models. The possible non-linearity of the relationships was explored by plotting residuals vs fitted values, assessing the Mean Absolute Percentage Errors, and goodness of fit (Bayesian Information Criterion, Akaike Information Criterion). These analyses indicated that the relationship between age and VO_2_ (peak or at usual pace) was approximately linear; whereas the energetic cost of walking exhibited a non-linear trend with age, with a steeper increase after the age of 65.

Linear regressions were performed to estimate the mean difference in MVO_2_ peak and aerobic resilience between mitochondrial function groups. Analyses were adjusted for age, sex, ethnicity, physical activity level and PCr percent depletion with exercise, considering their potential confounding role in the studied associations. Further regression models were evaluated after including multiplicative interactions between mitochondrial OxPhos level (categorised) and age. From the parameters of the regression equation, we estimated the intercept and slope values of the MVO_2_ peak and aerobic resilience versus OxPhos curves from younger to older age. Predicted values were shown in regression plots. Estimates were expressed as β-coefficient and 95% Confidence Intervals (95%CI) and were provided for the total sample and after sex-stratification. Concerning the energetic cost of walking, we used a regression spline function and tested the interaction between age (modelled with a B-spline with three degrees of freedom) and mitochondrial OxPhos groups after adjusting the model for the same covariates listed above.

To further explore the modifying effect of mitochondrial OxPhos on the relationship between age and MVO_2_ peak and aerobic resilience, we derived point estimates from sex-stratified linear regression models, including age, ethnicity, physical activity level, PCr percent depletion with exercise, k_PCr_ and the interaction age^*^k_PCr_. The mean or mode of quantitative or categorical covariates, respectively, was used to generate the point estimates.

As a sensitivity analysis, we performed quantile regression analysis to evaluate whether the differences in energetic metabolism variables by mitochondrial OxPhos depended on the outcome distribution. Moreover, linear regressions were repeated after categorising participants by sex-specific z-scores of k_PCr_.

All statistical tests were performed using R statistical software, and a *P*-value <.05 was considered statistically significant.

## Results

Characteristics of the 649 study participants are reported in Table 1. The mean age of the sample was 64.5 years (min-max 22–92, with 56.5% aged ≥65 years), 43.1% were males, and 24.5% were Black. The sample had high educational attainment of 17.7 mean years of schooling and cognitive performance with a mean MMSE score of 28.6, and 49.6% reported a moderate or very active lifestyle. When comparing sample characteristics based on mitochondrial OxPhos levels (Table 1), those with low values (k_PCr_ z-scores ≤ − 0.5) were more likely to be Black and physically inactive and to report fewer years of schooling and a positive history of diabetes, chronic heart failure, PAD and COPD than those with average or high OxPhos. MVO_2_ peak and the energetic cost of walking increased significantly from the low to the high mitochondrial OxPhos groups.

**Table 1:** Baseline characteristics of the total sample and by mitochondrial oxidative capacity levels

	**All (*n* = 649)**	**Mitochondrial OxPhos**			***P*-value**
		**Low (*n* = 229)**	**Average (*n* = 250)**	**High (*n* = 170)**	
Sex (male)	280 (43.1)	97 (42.4)	108 (43.2)	75 (44.1)	.94
Age (y)	64.48 (15.50)	64.37 (15.28)	64.74 (15.82)	64.25 (15.39)	.944
Ethnicity (Black)	159 (24.5)	88 (38.4)	43 (17.2)	28 (16.5)	<.001
Educational level (y)	17.65 (2.60)	17.24 (2.60)	17.73 (2.71)	18.09 (2.38)	.004
Physical activity					<.001
*Inactive*	53 (8.2)	29 (12.9)	18 (7.2)	6 (3.6)	
*Light*	271 (42.1)	110 (49.1)	109 (43.6)	52 (30.8)	
*Moderate*	198 (30.8)	58 (25.9)	80 (32.0)	60 (35.5)	
*Very active*	121 (18.8)	27 (12.1)	43 (17.2)	51 (30.2)	
Body Mass Index (kg/m^2^)	26.91 (4.65)	27.42 (4.81)	26.77 (4.45)	26.42 (4.67)	.131
MMSE total score	28.59 (1.43)	28.40 (1.56)	28.73 (1.38)	28.66 (1.30)	.053
Chronic diseases					
Anaemia	82 (14.9)	36 (18.6)	33 (15.3)	13 (9.2)	.056
Depression	49 (8.9)	19 (9.8)	24 (11.1)	6 (4.2)	.069
Cancer	61 (11.1)	29 (14.9)	18 (8.3)	14 (9.9)	.09
Hypertension	244 (44.2)	94 (48.5)	81 (37.5)	69 (48.6)	.039
Diabetes	51 (9.2)	28 (14.4)	16 (7.4)	7 (4.9)	.006
Chronic heart failure	35 (6.3)	19 (9.8)	11 (5.1)	5 (3.5)	.042
Stroke	49 (8.9)	21 (10.8)	21 (9.7)	7 (4.9)	.147
Peripheral artery disease	20 (3.6)	12 (6.2)	3 (1.4)	5 (3.5)	.034
Chronic Obstructive Pulmonary Disease	81 (14.7)	38 (19.6)	31 (14.4)	12 (8.5)	.017
Joint diseases	292 (52.9)	100 (51.5)	119 (55.1)	73 (51.4)	.71
Parkinson’s disease	3 (0.5)	0 (0.0)	3 (1.4)	0 (0.0)	.096
k_PCr_ (s^−1*^1000)	22.00 (5.81)	17.05 (2.30)	21.72 (2.68)	29.07 (5.28)	<.001
PCr depletion (%)	55.58 (11.69)	56.93 (11.58)	56.57 (11.73)	52.23 (11.17)	<.001
MVO_2_ peak (ml/kg/min)					
*Males*	26.74 (8.65)	24.4 (7.36)	26.2 (8.62)	30.5 (9.07)	<.001
*Females*	21.99 (6.39)	20.1 (5.73)	22.4 (5.87)	24.0 (7.28)	<.001
Energetic cost of walking (ml/kg/100 m)	15.50 (2.80)	14.81 (2.74)	15.88 (3.00)	15.82 (2.41)	<.001
Aerobic resilience					
*Males*	2.32 (0.77)	2.24 (0.70)	2.27 (0.74)	2.49 (0.85)	.093
*Females*	1.94 (0.57)	1.87 (0.53)	1.98 (0.58)	1.97 (0.60)	.305

*Notes*: Values are expressed as mean (standard deviations) for quantitative variables, and as count (%) for categorical variables. Mitochondrial OxPhos groups are categorised based on age- and sex-specific z-scores of k_PCr_ ≤ −0.5 (low), between −0.5 and 0.5 (average) and > 0.5 (high). *P*-values refer to the difference between mitochondrial OxPhos groups. Some data were missing in educational level (*n* = 1), physical activity (*n* = 7), body mass index (*n* = 106), MMSE (*n* = 108), chronic diseases (*n* = 102 for each of the listed diseases), energetic cost of walking (*n* = 85) and aerobic resilience (*n* = 80). Aerobic resilience is the ratio between MVO_2_ peak (ml/kg/min) and VO_2_ during usual walking (ml/kg/min). *Abbreviations*: MMSE, Mini-Mental State Examination; MVO_2_ peak, peak oxygen consumption; OxPhos, oxidative phosphorylation; VO_2_, oxygen consumption.

**Figure 1:**
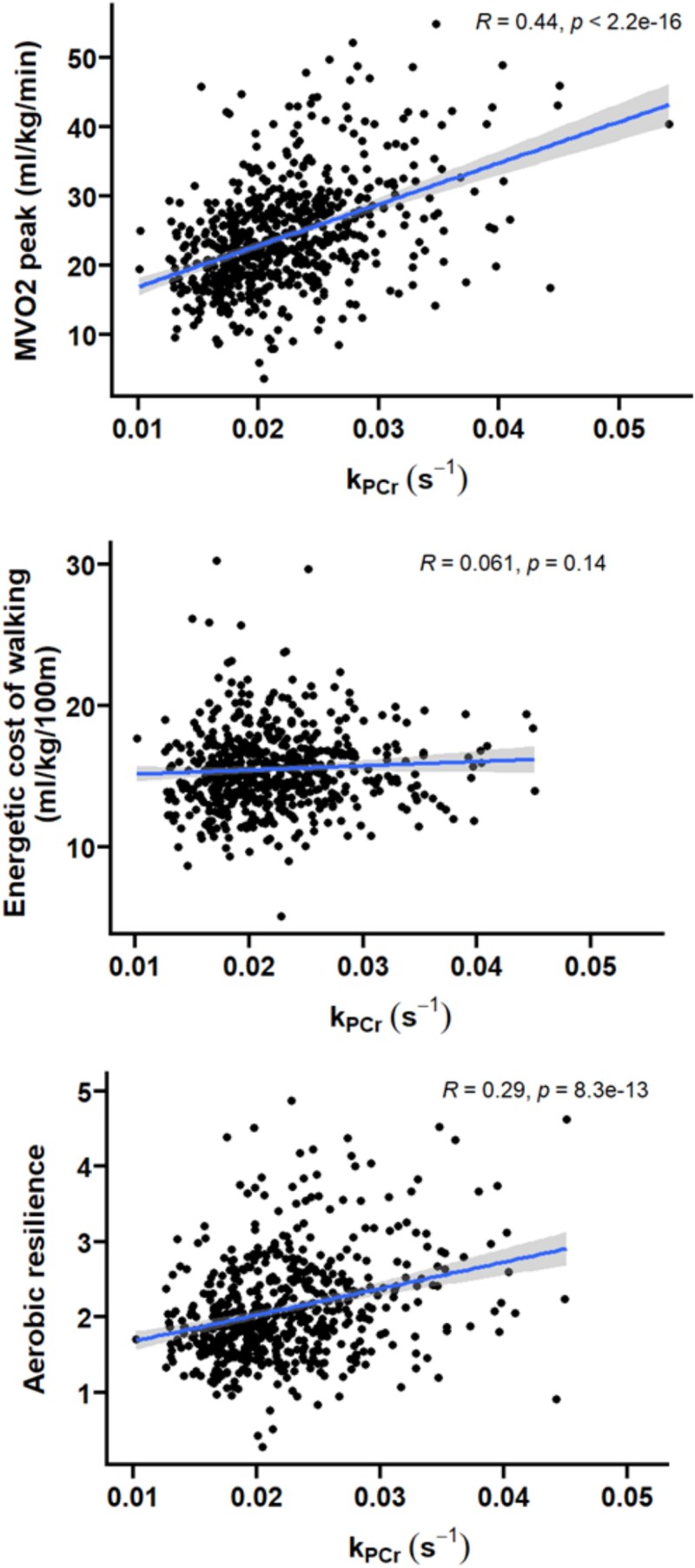
Scatterplot and correlation coefficient of k_PCr_ with peak oxygen consumption, the energetic cost of walking and aerobic resilience. *Abbreviations*: k_PCr_, phosphocreatine recovery rate; MVO_2_ peak, peak oxygen consumption.

In simple correlation analyses, mitochondrial OxPhos measured as k_PCr_ showed a positive correlation with MVO_2_ peak (*r* = 0.44, *P* < .001) and aerobic resilience (*r* = 0.29, *P* < .001), while there was no significant association with the energetic cost of walking (Figure 1).

**Figure 2:**
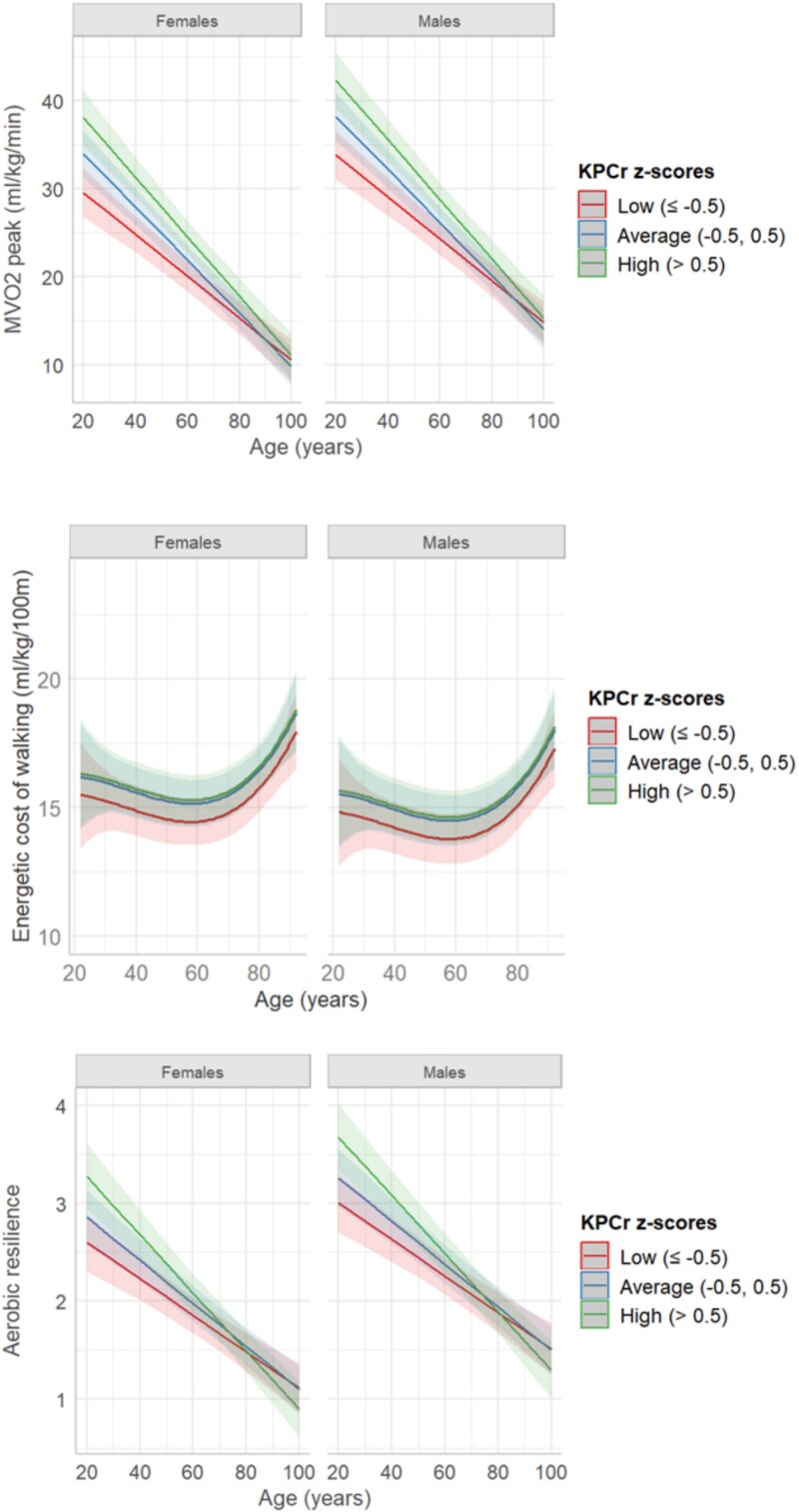
Predicted peak oxygen consumption, energetic cost of walking and aerobic resilience as a function of age, by sex and mitochondrial oxidative capacity levels. *Notes*: Plots illustrate predicted values from linear regression models adjusted for age, sex, ethnicity, physical activity level and PCr percent depletion with exercise. Analyses include 613 (for MVO_2_ peak) and 548 (for aerobic resilience) individuals with no missing data in the covariates. Mitochondrial oxidative capacity groups are categorised based on age- and sex-specific z-scores of k_PCr_ ≤ −0.5 (low), between −0.5 and 0.5 (average) and > 0.5 (high).

In multivariable linear regression analyses (Table 2), participants with average (k_PCr_ z-scores from −0.5 to 0.5 SD) or higher (k_PCr_ z-scores >0.5 SD) mitochondrial OxPhos had 1.59 (95%CI: 0.56, 2.63) and 4.07 mL/kg/min (95%CI: 2.88, 5.26) higher MVO_2_ peak, respectively, than the lower k_PCr_ group. Similarly, participants with high k_PCr_ had, on average, 0.19 higher aerobic resilience (95% CI: 0.06, 0.32) compared to those with low k_PCr_. Participants with average and high k_PCr_ showed energetic cost of walking values greater by 0.71 (95% CI 0.16, 1.26; *P* = .01) and 0.84 (95% CI: 0.21, 1.47; *P* = .009) ml/kg/100 m than those with low k_PCr_, respectively (data not shown). When stratifying the analyses by sex, we observed greater effect sizes for the associations related to MVO_2_ peak and aerobic resilience in males than females, with no significant sex differences identified in the findings regarding the energetic cost of walking (Table 2, Figure 2).

**Table 2:** Linear regression for the association of k_PCr_ with peak oxygen consumption and aerobic resilience stratified by sex

	**All**		**Males**		**Females**	
	**β (95%CI)**	***P*-value**	**β (95%CI)**	***P*-value**	**β (95%CI)**	***P*-value**
**Outcome: MVO**_**2**_ **peak (ml/kg/min)**						
Low k_PCr_	[ref]		[ref]		[ref]	
Average k_PCr_	**1.59 (0.56, 2.63)**	**.003**	**1.88 (0.07, 3.69)**	**.042**	**1.33 (0.16, 2.50)**	**.026**
High k_PCr_	**4.07 (2.88, 5.26)**	**<.001**	**5.42 (3.32, 7.51)**	**<.001**	**2.80 (1.45, 4.14)**	**<.001**
						
**Outcome: Aerobic resilience**						
Low k_PCr_	[ref]		[ref]		[ref]	
Average k_PCr_	0.11 (−0.00, 0.23)	.051	0.10 (−0.09, 0.29)	.291	0.12 (−0.02, 0.26)	.098
High k_PCr_	**0.19 (0.06, 0.32)**	**.004**	**0.28 (0.07, 0.50)**	**.011**	0.10 (−0.06, 0.26)	.215

*Notes*: Mitochondrial oxidative capacity groups are categorised based on age- and sex-specific z-scores of k_PCr_ ≤ −0.5 (low), between −0.5 and 0.5 (average) and > 0.5 (high). The linear regression model is adjusted for age, sex, ethnicity, physical activity level and PCr percent depletion with exercise. Analyses include 613 (for MVO_2_ peak) and 548 (for aerobic resilience) individuals with no missing data in the covariates. Bold values indicate statistically significant estimates. *Abbreviations*: MVO_2_ peak, peak oxygen consumption.

There was a significant interaction between age and k_PCr_ in regression models with MVO_2_ peak and aerobic resilience as dependent variables (for both models: *p_interaction_* = 0.01), but not in the regression analysis for the energetic cost of walking. Individuals with higher k_PCr_ had higher MVO_2_ peak and aerobic resilience from younger to older age, although the strength of this association substantially attenuated with increasing age, especially after 70 years (Figure 2 and **Appendix S4**  **in Supplementary Data)**. For example, examining point estimates of MVO_2_ peak and aerobic resilience by sex, age and mitochondrial OxPhos (see **Appendix S5**  **in Supplementary Data**), an individual at age 40 with low k_PCr_ had approximately the same MVO_2_ peak as a 60-year-old of the same sex but with high k_PCr_. This gap progressively narrowed with age and was less marked for aerobic resilience. Similar results were observed when performing quantile regression analyses (**Appendices S6**  **and**  **S7**, **Supplementary Data**), and when considering sex-specific z-scores of k_PCr_ (**Appendices S8**  **and**  **S9**, **Supplementary Data**).

## Discussion

Our study reinforces the hypothesis that muscle mitochondrial OxPhos plays a significant role in cardiorespiratory fitness and aerobic resilience from younger to older age. Individuals with greater mitochondrial OxPhos exhibited higher MVO_2_ peak and aerobic resilience across all age groups; however, this advantage appears to diminish with increasing age. In contrast, muscle mitochondrial OxPhos capacity does not appear to be substantially related to the energetic cost of walking across different ages.

Previous findings support a direct association between mitochondrial OxPhos and VO_2_ max in both young and older adults, with skeletal muscle mitochondrial energetics accounting for approximately one-third of the variability in VO_2_ max [11, 24]. Such a relationship is supported by several mechanisms. For instance, greater mitochondrial efficiency and capacity to restore PCr from inorganic phosphate (Pi) affect fatigability [12], which conditions an individual’s ability to undergo greater physical efforts. In addition, mitochondria are involved in diverse biological activities beyond OxPhos, such as regulating intracellular calcium homeostasis [25], apoptosis, and atrophic processes in skeletal muscle [26, 27], which could influence muscle quality and, indirectly, MVO_2_ peak. Alterations in mitochondrial efficiency reflect a higher production of reactive oxygen species, which promote the establishment of a chronic inflammatory status through multiple pathways [28, 29]. Moreover, mitochondrial dysfunction is linked to a massive release into the cytosol of damage-associated molecular species, including mtDNA (mainly in its oxidised forms) and cardiolipin, a structural inner-membrane phospholipid [30, 31]. Overall, these effects may have a central role in the development of sarcopenia and physical impairments [32, 33]. However, the contribution of these mechanisms to age-related changes in physical fitness observed in our study remains undetermined. In fact, whether OxPhos capacity correlates with other mitochondrial functions is unknown because no standard method exists to assess non-OxPhos mitochondrial function in humans.

Interestingly, the difference in MVO_2_ peak between individuals with varying levels of mitochondrial OxPhos gradually diminished with ageing. This gap was particularly pronounced in younger individuals, in whom a difference of one SD in mitochondrial OxPhos corresponded to a cardiorespiratory fitness of nearly 20 years older. Conversely, by the age of 70, the difference in fitness accounted for by differences in OxPhos was minimal, suggesting that additional factors other than mithocondrial oxidative capacity may have a strong influence on cardiorespiratory fitness with advancing age. Notably, these findings were derived from regression analyses adjusted for physical activity levels. This likely buffered the differences observed between groups since physical activity may be either a confounder (by influencing mitochondrial OxPhos and cardiorespiratory fitness [7]) or a mediator of the studied association since mitochondrial OxPhos may affect the level to which individuals can be engaged in exercise, especially in older age. A second hypothesis concerns the possible greater relevance of the decrease in muscle mass compared to mitochondrial energetics to older people’s MVO_2_ peak. This interpretation would contrast with previous studies that reported mitochondrial OxPhos reduction to be important and independent of the loss of muscle mass in determining age-related changes in VO_2_ max [34]. A third hypothesis is that the accumulation of deficits and chronic diseases associated with ageing may significantly impact peak MVO_2_ in the oldest individuals, thereby accounting for a large portion of the variability in cardiorespiratory fitness above and beyond the role played by mitochondrial OxPhos [35]. Moreover, besides the number of chronic diseases, individuals in the oldest age classes exhibit a higher frequency of cardiovascular, metabolic and respiratory conditions, which can substantially affect aerobic capacity [35]. Finally, participants in the oldest age groups who could perform the physical performance tests were likely the healthiest. Therefore, although age-specific z-scores of k_PCr_ should take into account the physiological decline of OxPhos with advancing age, selective survival could have mitigated the differential impact of mitochondrial OxPhos on the outcomes. Accordingly, the variability in k_PCr_ values with advancing age gradually reduced.

Similar results were observed for aerobic resilience, a measure obtained from the ratio of MVO_2_ peak to average VO_2_ during walking, which indicates the extent to which individuals can increase their aerobic metabolism from usual activities to maximal effort. In our sample, adults with higher mitochondrial OxPhos showed greater aerobic resilience; however, the difference in aerobic resilience by OxPhos level attenuated in older participants. This trend was likely driven by differences in MVO_2_ peak, as mitochondrial OxPhos did not appear to be substantially associated with the energetic cost of walking. In particular, the energetic cost of walking did not correlate with mitochondrial OxPhos, and only a minimal difference in this parameter (less than 0.01 mL/kg/m) emerged comparing individuals with average or high vs. low OxPhos. This result leaves room for various interpretations. On the one hand, the sample included individuals who were physically fit enough to undergo the treadmill test and could be relatively homogeneous in terms of energetic walking cost. In this context, differences in physical fitness may more markedly emerge when considering aerobic capacity during maximal effort than during usual activities. On the other hand, our findings might suggest that walking energetics is influenced more by the integrity and functionality of the biomechanical and kinesiological systems that optimise energy consumption during gait [22, 36, 37] than mitochondrial efficiency [38]. Concerning the former, changes in coordination, balance, and other walking mechanics’ features (e.g. stride length, joint ranges of motion, antagonist muscle co-activation timing) in older age may be attributed to common symptoms (e.g. osteoarticular pain) and multisystemic dysfunctions, including neurodegenerative processes. Accordingly, previous studies have demonstrated that certain biomarkers of neurodegeneration, such as brain and hippocampal atrophy, as well as amyloid-beta deposition, are inversely associated with the energetic cost of walking [39, 40].

Our analysis of sex-related differences revealed that the disparity in aerobic capacity and resilience between the higher and lower mitochondrial OxPhos groups was nearly twice as pronounced in males as in females. These results extend current knowledge by emphasising sex-specific patterns in the association between mitochondrial OxPhos and mobility performance [41]. Although some evidence suggests higher intrinsic mitochondrial function in females [42], other studies have found similar respiration rates in both sexes, but differences in mitochondrial membrane microviscosity, fatty acid oxidation and ADP sensitivity [41, 43, 44]. Moreover, males and females likely have differences in the biomechanics of walking, which may also help explain our results [45].

The strengths of our study include the relatively large sample of individuals across different age groups with assessments of mitochondrial OxPhos and maximal aerobic capacity using high-quality and validated approaches. Moreover, in addition to the MVO_2_ peak, measuring energy expenditure during walking at the preferred pace through indirect calorimetry allowed us to derive a measure of aerobic resilience, which represents a novelty of this work. On the other hand, possible limitations include the cross-sectional study design and the lower representation of individuals in the younger age group, which may have affected the statistical power of the analysis on age-related changes. Additionally, we acknowledge that the participants who underwent the treadmill test may not be representative of the general older population, particularly the frailest individuals and those with high-risk cardiovascular conditions, who did not take the test. Accordingly, the key characteristics of the study participants include high educational attainment, healthy lifestyle and preserved cognitive function.

In conclusion, our study underscores the critical role of mitochondrial OxPhos in elucidating the differential age-related decline in aerobic capacity. However, our findings also suggest that, in older age, other factors beyond mitochondrial OxPhos become increasingly influential in determining fitness and play a vital role in enhancing the capacity for aerobic metabolism under greater exertion. Reduced physical fitness is a driving determinant of physical performance impairment and loss of functional ability, which may lead to the development of disability and the need for higher assistance and healthcare services. Larger studies with a longitudinal design are required to further verify the role of muscle mitochondrial OxPhos and deepen our understanding of the additional factors that modulate individual functional trajectories from adult to older age. Moreover, these insights emphasise the importance of investigating the effectiveness of multifactorial interventions targeted but not be focused exclusively to mitochondrial efficiency, to mitigate the detrimental effects of ageing on physical function and self-sufficiency.

## Supplementary Material

afag022_Supplementary_materials

## References

[1] Nguyen QD, Moodie EM, Forget MF et al. Health heterogeneity in older adults: exploration in the Canadian longitudinal study on aging. J Am Geriatr Soc 2021;69:678–87. 10.1111/jgs.16919. 33155270

[2] Studenski S, Perera S, Patel K et al. Gait speed and survival in older adults. JAMA 2011;305:50–8. 10.1001/jama.2010.1923. 21205966 PMC3080184

[3] WHO . *What Is Healthy Ageing?* World Health Organization, Geneva, Switzerland, 2018.

[4] Tian Q, Mitchell BA, Zampino M et al. Muscle mitochondrial energetics predicts mobility decline in well-functioning older adults: the Baltimore longitudinal study of aging. Aging Cell 2022;21:e13552. 10.1111/acel.13552. 35048491 PMC8844110

[5] Tian Q, Bilgel M, Walker KA et al. Skeletal muscle mitochondrial function predicts cognitive impairment and is associated with biomarkers of Alzheimer's disease and neurodegeneration. Alzheimers Dement 2023;19:4436–45. 10.1002/alz.13388. 37530130 PMC10592411

[6] Conley KE, Jubrias SA, Esselman PC. Oxidative capacity and ageing in human muscle. J Physiol 2000;526 Pt 1:203–10. 10.1111/j.1469-7793.2000.t01-1-00203.x. 10878112 PMC2269983

[7] Grevendonk L, Connell NJ, McCrum C et al. Impact of aging and exercise on skeletal muscle mitochondrial capacity, energy metabolism, and physical function. Nat Commun 2021;12:4773. 10.1038/s41467-021-24956-2. 34362885 PMC8346468

[8] Tian Q, Lee PR, Walker KA et al. Energizing mitochondria to prevent mobility loss in aging: rationale and hypotheses. Exerc Sport Sci Rev 2023;51:96–102. 10.1249/jes.0000000000000315. 37057904 PMC10258139

[9] Schrack JA, Simonsick EM, Ferrucci L. The energetic pathway to mobility loss: An emerging new framework for longitudinal studies on aging. J Am Geriatr Soc 2010;58 Suppl 2:S329–36. 10.1111/j.1532-5415.2010.02913.x. 21029063 PMC3057770

[10] Schrack JA, Simonsick EM, Chaves PH et al. The role of energetic cost in the age-related slowing of gait speed. J Am Geriatr Soc 2012;60:1811–6. 10.1111/j.1532-5415.2012.04153.x. 23035640 PMC3470763

[11] Mau T, Lui LY, Distefano G et al. Mitochondrial energetics in skeletal muscle are associated with leg power and cardiorespiratory fitness in the study of muscle, mobility and aging. J Gerontol A Biol Sci Med Sci 2023;78:1367–75. 10.1093/gerona/glac238. 36462195 PMC10395564

[12] Santanasto AJ, Glynn NW, Jubrias SA et al. Skeletal muscle mitochondrial function and fatigability in older adults. J Gerontol A Biol Sci Med Sci 2015;70:1379–85. 10.1093/gerona/glu134. 25167867 PMC4612379

[13] Zane AC, Reiter DA, Shardell M et al. Muscle strength mediates the relationship between mitochondrial energetics and walking performance. Aging Cell 2017;16:461–8. 10.1111/acel.12568. 28181388 PMC5418194

[14] Mau T, Barnes HN, Blackwell TL et al. Lower muscle mitochondrial energetics is associated with greater phenotypic frailty in older women and men: the study of muscle, mobility and aging. Geroscience 2024;46:2409–24. 10.1007/s11357-023-01002-1. 37987886 PMC10828481

[15] Qiao YS, Santanasto AJ, Coen PM et al. Associations between skeletal muscle energetics and accelerometry-based performance fatigability: study of muscle, mobility and aging. Aging Cell 2024;23:e14015. 10.1111/acel.14015. 37843879 PMC11166367

[16] Strasser B, Burtscher M. Survival of the fittest: VO(2)max, a key predictor of longevity? Front Biosci (Landmark Ed) 2018;23:1505–16. 10.2741/4657. 29293447

[17] National Institute on Aging . The Baltimore Longitudinal Study of Aging. https://www.nia.nih.gov/research/labs/blsa

[18] Ferrucci L . The Baltimore longitudinal study of aging (BLSA): a 50-year-long journey and plans for the future. J Gerontol A Biol Sci Med Sci 2008;63:1416–9. 10.1093/gerona/63.12.1416. 19126858 PMC5004590

[19] Choi S, Reiter DA, Shardell M et al. 31P magnetic resonance spectroscopy assessment of muscle bioenergetics as a predictor of gait speed in the Baltimore longitudinal study of aging. J Gerontol A 2016;71:1638–45. 10.1093/gerona/glw059. PMC5106855 27075894

[20] Cohen J . *Statistical Power Analysis for the Behavioral Sciences*. 2nd edition. Routledge, New York, 1988.

[21] Schrack JA, Zipunnikov V, Simonsick EM et al. Rising energetic cost of walking predicts gait speed decline with aging. J Gerontol A Biol Sci Med Sci 2016;71:947–53. 10.1093/gerona/glw002. 26850913 PMC4906328

[22] Brown C, Simonsick E, Schrack J et al. Impact of balance on the energetic cost of walking and gait speed. J Am Geriatr Soc 2023;71:3489–97. 10.1111/jgs.18521. 37528742 PMC12159796

[23] Dougherty RJ, Wanigatunga AA, An Y et al. Walking energetics and white matter hyperintensities in mid-to-late adulthood. Alzheimers Dement (Amst) 2023;15:e12501. 10.1002/dad2.12501. 38026756 PMC10646278

[24] Coen PM, Jubrias SA, Distefano G et al. Skeletal muscle mitochondrial energetics are associated with maximal aerobic capacity and walking speed in older adults. J Gerontol A Biol Sci Med Sci 2013;68:447–55. 10.1093/gerona/gls196. 23051977 PMC3593613

[25] Olsson K, Cheng AJ, Al-Ameri M et al. Impaired sarcoplasmic reticulum Ca(2+) release is the major cause of fatigue-induced force loss in intact single fibres from human intercostal muscle. J Physiol 2020;598:773–87. 10.1113/jp279090. 31785106

[26] Marzetti E, Wohlgemuth SE, Lees HA et al. Age-related activation of mitochondrial caspase-independent apoptotic signaling in rat gastrocnemius muscle. Mech Ageing Dev 2008;129:542–9. 10.1016/j.mad.2008.05.005. 18579179 PMC2585824

[27] Kubat GB, Bouhamida E, Ulger O et al. Mitochondrial dysfunction and skeletal muscle atrophy: causes, mechanisms, and treatment strategies. Mitochondrion 2023;72:33–58. 10.1016/j.mito.2023.07.003. 37451353

[28] Walker KA, Basisty N, Wilson DM 3rd. et al. Connecting aging biology and inflammation in the omics era. J Clin Invest 2022;132:e158448. 10.1172/jci158448. 35838044 PMC9282936

[29] He M, Chiang HH, Luo H et al. An acetylation switch of the NLRP3 inflammasome regulates aging-associated chronic inflammation and insulin resistance. Cell Metab 2020;31:580–591.e5. 10.1016/j.cmet.2020.01.009. 32032542 PMC7104778

[30] Jang JY, Blum A, Liu J et al. The role of mitochondria in aging. J Clin Invest 2018;128:3662–70. 10.1172/JCI120842. 30059016 PMC6118639

[31] Galluzzi L, Vanpouille-Box C. BAX and BAK at the gates of innate immunity. Trends Cell Biol 2018;28:343–5. 10.1016/j.tcb.2018.02.010. 29555208

[32] Sayed RKA, Fernández-Ortiz M, Diaz-Casado ME et al. Lack of NLRP3 inflammasome activation reduces age-dependent sarcopenia and mitochondrial dysfunction, favoring the prophylactic effect of melatonin. J Gerontol A 2019;74:1699–708. 10.1093/gerona/glz079. 30869745

[33] Pan L, Xie W, Fu X et al. Inflammation and sarcopenia: a focus on circulating inflammatory cytokines. Exp Gerontol 2021;154:111544. 10.1016/j.exger.2021.111544. 34478826

[34] Conley KE, Esselman PC, Jubrias SA et al. Ageing, muscle properties and maximal O_2_ uptake rate in humans. J Physiol 2000;526 (Pt 1):211–7. 10.1111/j.1469-7793.2000.00211.x. 10878113 PMC2270003

[35] Hong SN, Lai FTT, Wang B et al. Age-specific multimorbidity patterns and burden on all-cause mortality and public direct medical expenditure: a retrospective cohort study. J Epidemiol Global Health 2024;14:1077–88. 10.1007/s44197-024-00256-y. PMC11444029 38869775

[36] Faraji S, Wu AR, Ijspeert AJ. A simple model of mechanical effects to estimate metabolic cost of human walking. Sci Rep 2018;8:10998. 10.1038/s41598-018-29429-z. 30030539 PMC6054663

[37] Boyer KA, Hayes KL, Umberger BR et al. Age-related changes in gait biomechanics and their impact on the metabolic cost of walking: report from a National Institute on Aging workshop. Exp Gerontol 2023;173:112102. 10.1016/j.exger.2023.112102. 36693530 PMC10008437

[38] Peyré-Tartaruga LA, Dewolf AH, di Prampero PE et al. Mechanical work as a (key) determinant of energy cost in human locomotion: recent findings and future directions. Exp Physiol 2021;106:1897–908. 10.1113/ep089313. 34197674

[39] Dougherty RJ, Ramachandran J, Liu F et al. Association of walking energetics with amyloid beta status: findings from the Baltimore longitudinal study of aging. Alzheimers Dement (Amst) 2021;13:e12228. 10.1002/dad2.12228. 34458552 PMC8377776

[40] Dougherty RJ, Liu F, An Y et al. Energetic cost of walking and brain atrophy in mid-to-late life. J Gerontol A Biol Sci Med Sci 2022;77:2068–76. 10.1093/gerona/glab309. 34628503 PMC9536456

[41] Junker A, Wang J, Gouspillou G et al. Human studies of mitochondrial biology demonstrate an overall lack of binary sex differences: a multivariate meta-analysis. FASEB J 2022;36:e22146. 10.1096/fj.202101628R. 35073429 PMC9885138

[42] Cardinale DA, Larsen FJ, Schiffer TA et al. Superior intrinsic mitochondrial respiration in women than in men. Front Physiol 2018;9:1133. 10.3389/fphys.2018.01133. PMC6108574 30174617

[43] Miotto PM, McGlory C, Holloway TM et al. Sex differences in mitochondrial respiratory function in human skeletal muscle. Am J Physiol Regul Integr Comp Physiol 2018;314:R909–15. 10.1152/ajpregu.00025.2018. 29513564 PMC6032304

[44] Torres MJ, Kew KA, Ryan TE et al. 17 beta-estradiol directly lowers mitochondrial membrane microviscosity and improves bioenergetic function in skeletal muscle. Cell Metab 2018;27:167–179.e7. 10.1016/j.cmet.2017.10.003. 29103922 PMC5762397

[45] Osawa Y, Studenski SA, Ferrucci L. Knee extension rate of velocity development affects walking performance differently in men and women. Exp Gerontol 2018;112:63–7. 10.1016/j.exger.2018.09.005. 30218708 PMC6221187

