# Supplementary material for: Association of mitochondrial oxidative capacity with physical fitness in ageing: the Baltimore longitudinal study of ageing
Source: Age Ageing. 2026 Feb 6;55(2):afag022. doi: 10.1093/ageing/afag022 (PMC13366268; doi:10.1093/ageing/afag022)
Supplement: afag022_Supplementary_materials [file afag022_supplementary_materials.docx]

**Association of Mitochondrial Oxidative Capacity with Physical Fitness in Aging: The Baltimore Longitudinal Study of Aging**

**SUPPLEMENTARY MATERIAL**

**Appendix 1. Details on sociodemographic, lifestyle, and medical information collection**

*Sociodemographic, lifestyle, and medical information.* For each participant, age, sex, and ethnicity were ascertained via self-report. Physical activity was assessed through a structured questionnaire that queried the frequency and type of activity performed, which was categorized as inactive (mostly sedentary, with some walking), light (engagement for 2-4 h/week in low-intensity exercise), moderate (engagement for ≥5 h/week in low-intensity exercise or for ≥3 h/week in medium intensity exercise) or very active (engagement for ≥5 h/week in medium-to-high- intensity exercise)(1). Body mass index was computed as weight in kilograms divided by squared height in meters (kg/m^2^). The presence of chronic conditions was based on a review of clinical, laboratory, and radiologic examinations, and medical and hospital records. In particular, the presence of chronic heart failure, peripheral artery disease (PAD), chronic obstructive pulmonary disease (COPD), diabetes mellitus, cancer, and lower limb osteoarthritis was recorded. Cognitive performance was assessed through the Mini-Mental State Examination(2).

**Appendix 2. Details on the mitochondrial oxidative capacity assessment**

Consistent with previous studies in the BLSA, mitochondrial OxPhos was assessed by the phosphocreatine (PCr) recovery rate. Briefly, participants underwent ^31^P MRS scanning on the quadriceps muscles of the left leg in a 3T Achieva MRI scanner (Philips, Best, The Netherlands) to quantify phosphorous-containing metabolites(3). After being positioned supine with a 30° knee flexion, participants were instructed to perform a rapid ballistic knee extension to consume the muscle’s PCr reservoir and maximize oxidative phosphorylation with minimal pH reduction. Before, during, and after the exercise, 75 pulse-acquire MRS spectra were obtained for a total of 7.5 min. The performed exercise did not exceed 42 s. Time-domain spectra analysis was performed using the jMRUI package (version 5.2) with the AMARES algorithm(4, 5).

Skeletal muscle oxidative ATP resynthesis was evaluated from the post-exercise PCr recovery rate. Specifically, PCr peak areas following exercise were fitted to the following mono-exponential function:

PCr_(t)_ = PCr_(t0)_ + ΔPCr * (1 – e^(-t*kPCr)^),

where PCr_(t0)_ is the PCr peak area at the end of the exercise (i.e., PCr recovery start), ΔPCr is the PCr peak area during the exercise, and k_PCr_ is the PCr recovery rate(3, 6, 7). Only tests resulting in a PCr reduction of at least 33% from the beginning to the end of exercise were considered valid, in line with previous studies(3, 6, 7).

**REFERENCES**

1. Moore AZ, Simonsick EM, Landman B, et al.; Correlates of life course physical activity in participants of the Baltimore Longitudinal Study of Aging. *Aging Cell* 2024;**23**(4):e14078. doi: 10.1111/acel.14078.

2. Folstein MF, Folstein SE, McHugh PR; Mini-mental state . A practical method for grading the cognitive state of patients for the clinician. *Journal of Psychiatric Research* 1975;**12**(3):189-198. doi: 10.1016/0022-3956(75)90026-6.

3. Choi S, Reiter DA, Shardell M, et al.; 31P Magnetic Resonance Spectroscopy Assessment of Muscle Bioenergetics as a Predictor of Gait Speed in the Baltimore Longitudinal Study of Aging. *The Journals of Gerontology: Series A* 2016;**71**(12):1638-1645. doi: 10.1093/gerona/glw059.

4. Vanhamme L, Van Huffel S, Van Hecke P, et al.; Time-domain quantification of series of biomedical magnetic resonance spectroscopy signals. *J Magn Reson* 1999;**140**(1):120-30. doi: 10.1006/jmre.1999.1835.

5. Naressi A, Couturier C, Devos JM, et al.; Java-based graphical user interface for the MRUI quantitation package. *MAGMA* 2001;**12**(2-3):141-52. doi: 10.1007/bf02668096.

6. Zane AC, Reiter DA, Shardell M, et al.; Muscle strength mediates the relationship between mitochondrial energetics and walking performance. *Aging Cell* 2017;**16**(3):461-468. doi: 10.1111/acel.12568.

7. Tian Q, Mitchell BA, Zampino M, et al.; Muscle mitochondrial energetics predicts mobility decline in well-functioning older adults: The Baltimore Longitudinal Study of Aging. *Aging Cell* 2022;**21**(2):e13552. doi: 10.1111/acel.13552.

**Appendix 3. Distribution of age-specific z-scores of k_PCr_ in the total sample**


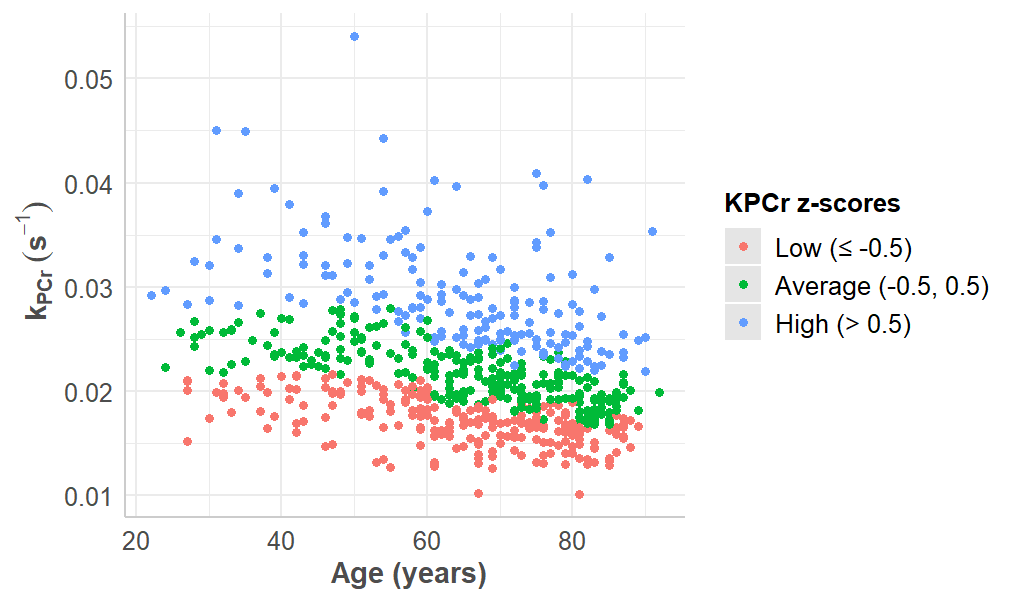


*Notes*. Mitochondrial oxidative capacity groups are categorized based on age- and sex-specific z-scores of k_PCr_ ≤ -0.5 (low), between -0.5 and 0.5 (average), and >0.5 (high).

**Appendix 4. Linear regression for the association of k_PCr_ with peak oxygen consumption and aerobic resilience in the total sample and stratified by sex**

|  | **All** | | **Males** | | **Females** | |
| --- | --- | --- | --- | --- | --- | --- |
|  | **ẞ (95%CI)** | **p-value** | **ẞ (95%CI)** | **p-value** | **ẞ (95%CI)** | **p-value** |
| **Outcome: MVO2 peak** |  |  |  |  |  |  |
| *Intercept* |  |  |  |  |  |  |
| Low k_PCr_ | [ref] |  | [ref] |  | [ref] |  |
| Average k_PCr_ | **5.65 (1.40, 9.90)** | **0.009** | **7.99 (0.78, 15.21)** | **0.030** | 4.15 (-0.77, 9.07) | 0.098 |
| High k_PCr_ | **10.48 (5.63, 15.32)** | **<0.001** | **11.90 (4.01, 19.79)** | **0.003** | **8.79 (2.90, 14.68)** | **0.004** |
| *Interaction with age* |  |  |  |  |  |  |
| Average k_PCr_ | -0.06 (-0.13, 0.00) | 0.053 | -0.10 (-0.20, 0.01) | 0.085 | -0.04 (-0.12, 0.03) | 0.247 |
| High k_PCr_ | **-0.10 (-0.17, -0.03)** | **0.008** | -0.10 (-0.22, 0.02) | **0.095** | -0.09 (-0.18, -0.01) | 0.04 |
|  |  |  |  |  |  |  |
| **Outcome: aerobic resilience** |  |  |  |  |  |  |
| *Intercept* |  |  |  |  |  |  |
| Low k_PCr_ | [ref] |  | [ref] |  | [ref] |  |
| Average k_PCr_ | 0.32 (-0.13, 0.78) | 0.165 | 0.35 (-0.39, 1.08) | 0.353 | 0.35 (-0.22, 0.92) | 0.226 |
| High k_PCr_ | **0.89 (0.37, 1.42)** | **0.001** | **1.18 (0.37, 1.99)** | **0.005** | 0.58 (-0.10, 1.26) | 0.092 |
| *Interaction with age* |  |  |  |  |  |  |
| Average k_PCr_ | -0.003 (-0.01, 0.003) | 0.346 | -0.004 (-0.02, 0.01) | 0.486 | -0.004 (-0.01, 0.01) | 0.405 |
| High k_PCr_ | **-0.01 (-0.02, -0.003)** | **0.007** | **-0.01 (-0.03, -0.002)** | **0.025** | -0.01 (-0.02, 0.003) | 0.150 |

*Notes*. Mitochondrial oxidative capacity groups are categorized based on age- and sex-specific z-scores of k_PCr_ ≤ -0.5 (low), between -0.5 and 0.5 (average), and >0.5 (high). The linear regression model is adjusted for age, sex, ethnicity, physical activity level, and PCr percent depletion with exercise. Analyses include 613 (for MVO2 peak) and 548 (for aerobic resilience) individuals with no missing data in the covariates. Bold values indicate statistically significant estimates. *Abbreviations*: MVO2 peak, peak oxygen consumption.

**Appendix 5. Point estimates of peak oxygen consumption and aerobic resilience by sex, age and mitochondrial oxidative capacity levels**

|  |  | **Mitochondrial OxPhos** | | |
| --- | --- | --- | --- | --- |
|  | **Age (years)** | **Low** | **Average** | **High** |
| **MVO2 peak (ml/kg/min)** | |  |  |  |
| Males | 20 | 33.2 (30.8, 35.6) | 37.6 (35.2, 40) | 41.7 (38.8, 44.5) |
|  | 40 | 28.5 (26.8, 30.1) | 31.6 (29.8, 33.3) | 34.9 (32.9, 36.9) |
|  | 60 | 23.7 (22.5, 24.9) | 25.5 (24.2, 26.9) | 28.2 (26.7, 29.7) |
|  | 80 | 19.0 (17.5, 20.4) | 19.5 (18.0, 21.0) | 21.4 (19.7, 23.1) |
| Females | 20 | 29.0 (26.6, 31.3) | 33.3 (31.0, 35.6) | 37.4 (34.6, 40.3) |
|  | 40 | 24.2 (22.6, 25.8) | 27.3 (25.7, 28.9) | 30.7 (28.8, 32.6) |
|  | 60 | 19.5 (18.4, 20.6) | 21.3 (20.1, 22.5) | 23.9 (22.5, 25.3) |
|  | 80 | 14.7 (13.4, 16.0) | 15.3 (13.9, 16.7) | 17.2 (15.6, 18.8) |
| **Aerobic resilience** | |  |  |  |
| Males | 20 | 3.1 (2.8, 3.4) | 3.4 (3.1, 3.6) | 3.8 (3.5, 4.1) |
|  | 40 | 2.7 (2.6, 2.9) | 2.9 (2.7, 3.1) | 3.2 (3.0, 3.4) |
|  | 60 | 2.4 (2.2, 2.5) | 2.5 (2.3, 2.6) | 2.6 (2.4, 2.8) |
|  | 80 | 2.0 (1.8, 2.1) | 2.0 (1.9, 2.2) | 2.0 (1.8, 2.2) |
| Females | 20 | 2.7 (2.5, 3.0) | 3.0 (2.7, 3.2) | 3.4 (3.1, 3.7) |
|  | 40 | 2.3 (2.2, 2.5) | 2.5 (2.3, 2.7) | 2.8 (2.6, 3.0) |
|  | 60 | 2.0 (1.8, 2.1) | 2.1 (1.9, 2.2) | 2.2 (2.0, 2.3) |
|  | 80 | 1.6 (1.4, 1.7) | 1.6 (1.5, 1.8) | 1.6 (1.4, 1.8) |

*Notes*. Mitochondrial oxidative capacity groups are categorized based on age- and sex-specific z-scores of k_PCr_ ≤ -0.5 (low), between -0.5 and 0.5 (average), and >0.5 (high). Point estimates are derived from linear regression models including age, ethnicity, physical activity level, PCr percent depletion with exercise, k_PCr_, and the interaction age*k_PCr_. The mean or mode of quantitative or categorical variables, respectively, were used to generate the point estimates. *Abbreviations*: OxPhos, oxidative phosphorylation; MVO2 peak, peak oxygen consumption.

**Appendix 6. 10^th^, 50^th^, and 90^th^ percentile distribution of peak oxygen consumption by age and mitochondrial oxidative capacity levels**


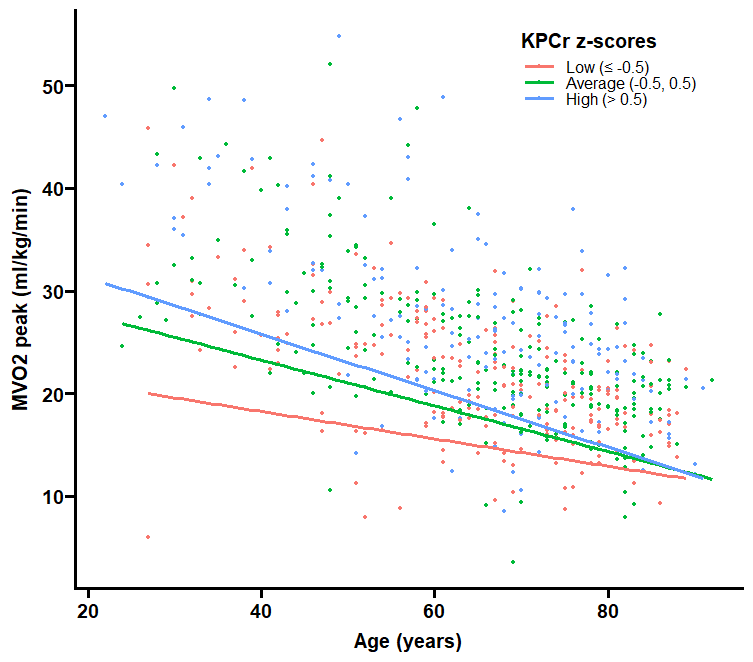


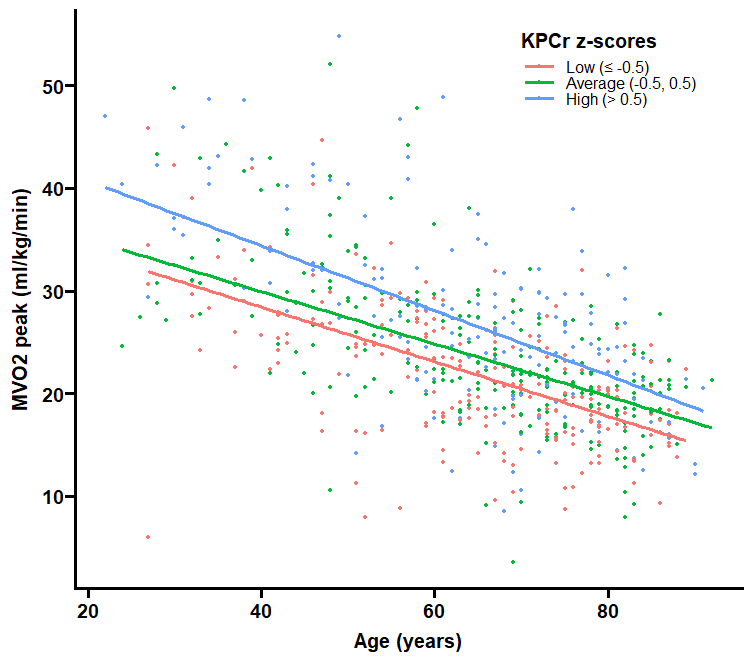


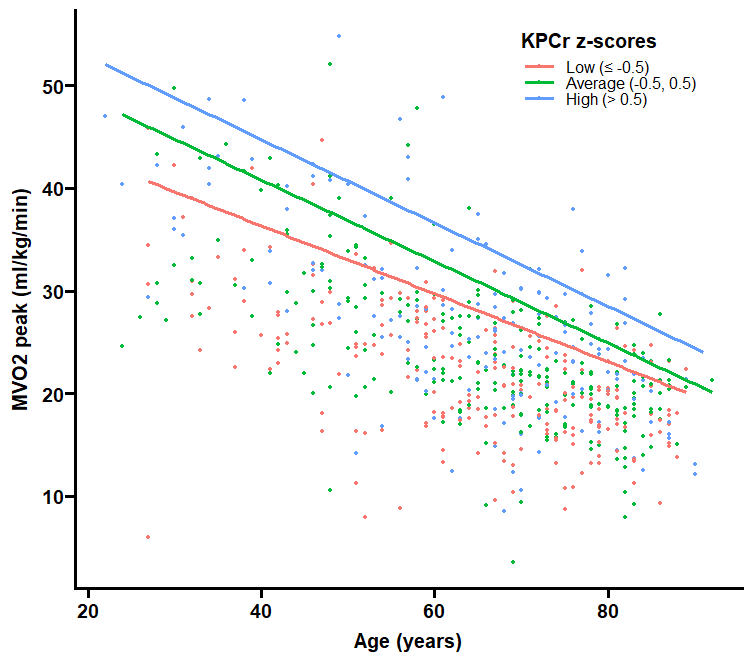


*Notes*. Mitochondrial oxidative capacity groups are categorized based on age- and sex-specific z-scores of k_PCr_ ≤ -0.5 (low), between -0.5 and 0.5 (average), and >0.5 (high). *Abbreviations*: MVO2 peak, peak oxygen consumption.

**Appendix 7. 10^th^, 50^th^, and 90^th^ percentile distribution of aerobic resilience by age and mitochondrial oxidative capacity levels**

**
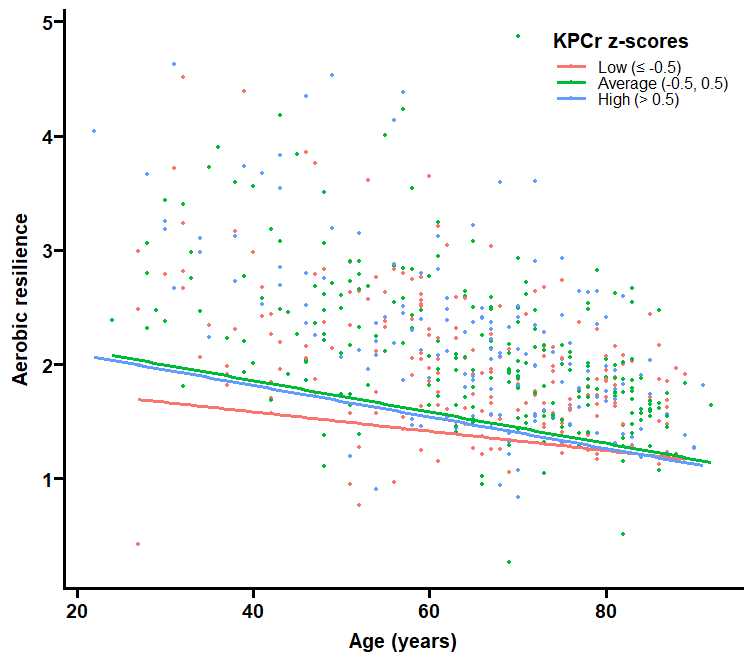
**

**
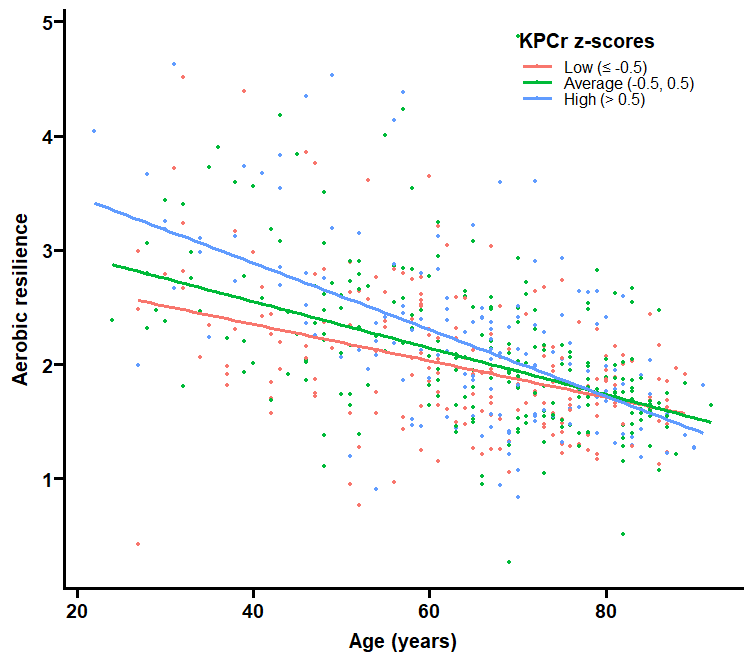
**

**
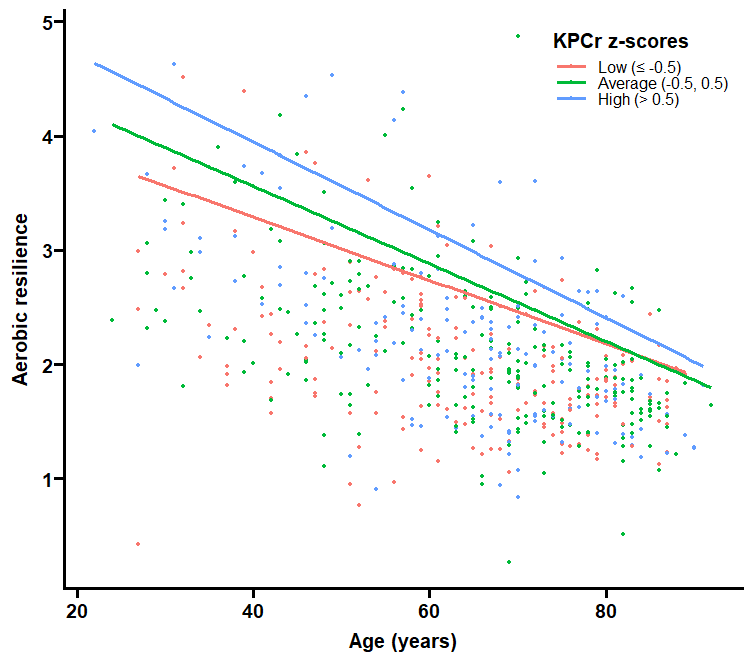
**

*Notes*. Mitochondrial oxidative capacity groups are categorized based on age- and sex-specific z-scores of k_PCr_ ≤ -0.5 (low), between -0.5 and 0.5 (average), and >0.5 (high).

**Appendix 8. Linear regression for the association of k_PCr_ (considering sex-specific z-scores) with peak oxygen consumption and aerobic resilience stratified by sex**

|  | **All** | | **Males** | | **Females** | |
| --- | --- | --- | --- | --- | --- | --- |
|  | **ẞ (95%CI)** | **p-value** | **ẞ (95%CI)** | **p-value** | **ẞ (95%CI)** | **p-value** |
| **Outcome: MVO2 peak (ml/kg/min)** |  |  |  |  |  |  |
| Low k_PCr_ | [ref] |  | [ref] |  | [ref] |  |
| Average k_PCr_ | 0.92 (-0.14, 1.98) | **0.088** | 0.31 (-1.49, 2.10) | 0.737 | **1.43 (0.23, 2.63)** | **0.02** |
| High k_PCr_ | **3.80 (2.53, 5.07)** | **<0.001** | **5.99 (3.80, 8.18)** | **<0.001** | **2.33 (0.90, 3.76)** | **0.002** |
|  |  |  |  |  |  |  |
| **Outcome: Aerobic resilience** |  |  |  |  |  |  |
| Low k_PCr_ | [ref] |  | [ref] |  | [ref] |  |
| Average k_PCr_ | 0.08 (-0.03, 0.20) | 0.155 | 0.004 (-0.18, 0.19) | 0.970 | **0.16 (0.02, 0.30)** | **0.029** |
| High k_PCr_ | **0.18 (0.04, 0.31)** | **0.012** | **0.30 (0.07, 0.53)** | **0.01** | 0.11 (-0.05, 0.28) | 0.173 |

*Notes*. Mitochondrial oxidative capacity groups are categorized based on sex-specific z-scores of k_PCr_ ≤ -0.5 (low), between -0.5 and 0.5 (average), and >0.5 (high). The linear regression model is adjusted for age, sex, ethnicity, physical activity level, and PCr percent depletion with exercise. Analyses include 613 (for MVO2 peak) and 548 (for aerobic resilience) individuals with no missing data in the covariates. Bold values indicate statistically significant estimates. *Abbreviations*: MVO2 peak, peak oxygen consumption.

**Appendix 9. Point estimates of peak oxygen consumption and aerobic resilience by sex, age and mitochondrial oxidative capacity levels (considering sex-specific z-scores of k_PCr_)**

|  |  | **Mitochondrial OxPhos** | | |
| --- | --- | --- | --- | --- |
|  | **Age (years)** | **Low** | **Average** | **High** |
| **MVO2 peak (ml/kg/min)** | |  |  |  |
| Males | 20 | 32.3 (29.0, 35.5) | 35.5 (33.2, 37.7) | 39.4 (36.8, 41.9) |
|  | 40 | 27.9 (25.8, 30.1) | 30.2 (28.6, 31.9) | 33.6 (31.8, 35.4) |
|  | 60 | 23.6 (22.3, 25.0) | 25 (23.7, 26.3) | 27.8 (26.3, 29.3) |
|  | 80 | 19.3 (17.9, 20.7) | 19.8 (18.2, 21.3) | 22.0 (20.1, 24.0) |
| Females | 20 | 27.9 (24.7, 31.1) | 31.1 (28.9, 33.3) | 35.0 (32.5, 37.4) |
|  | 40 | 23.6 (21.5, 25.7) | 25.8 (24.3, 27.4) | 29.2 (27.5, 30.9) |
|  | 60 | 19.2 (18.0, 20.5) | 20.6 (19.4, 21.8) | 23.4 (22.1, 24.8) |
|  | 80 | 14.9 (13.6, 16.2) | 15.4 (14, 16.8) | 17.7 (15.8, 19.5) |
| **Aerobic resilience** | |  |  |  |
| Males | 20 | 2.9 (2.6, 3.3) | 3.4 (3.1, 3.6) | 3.5 (3.3, 3.8) |
|  | 40 | 2.6 (2.4, 2.8) | 2.9 (2.7, 3.1) | 3.1 (2.9, 3.2) |
|  | 60 | 2.3 (2.2, 2.5) | 2.5 (2.3, 2.6) | 2.6 (2.4, 2.7) |
|  | 80 | 2.0 (1.9, 2.2) | 2.0 (1.9, 2.2) | 2.1 (1.8, 2.3) |
| Females | 20 | 2.5 (2.2, 2.8) | 3.0 (2.7, 3.2) | 3.1 (2.9, 3.4) |
|  | 40 | 2.2 (2.0, 2.4) | 2.5 (2.3, 2.7) | 2.6 (2.5, 2.8) |
|  | 60 | 1.9 (1.8, 2.0) | 2.1 (1.9, 2.2) | 2.1 (2.0, 2.3) |
|  | 80 | 1.6 (1.5, 1.8) | 1.6 (1.5, 1.8) | 1.6 (1.4, 1.9) |

*Notes*. Mitochondrial oxidative capacity groups are categorized based on sex-specific z-scores of k_PCr_ ≤ -0.5 (low), between -0.5 and 0.5 (average), and >0.5 (high). Point estimates are derived from linear regression models including age, ethnicity, physical activity level, drop in PCr peak area with exercise, k_PCr_, and the interaction age*k_PCr_. The mean or mode of quantitative or categorical variables, respectively, was used to generate the point estimates. *Abbreviations*: OxPhos, oxidative phosphorylation; MVO2 peak, peak oxygen consumption.
